# Preliminary Study on the Restoration of the Phospholipid Profile in Serum from Patients with COVID-19 by Treatment with Vitamin E

**DOI:** 10.3390/cimb46070429

**Published:** 2024-07-08

**Authors:** María Elena Soto, Linaloe Manzano-Pech, Verónica Guarner-Lans, Adrían Palacios-Chavarría, Rafael Ricardo Valdez-Vázquez, Raúl Martínez-Memije, Mohammed El-Hafidi, Félix Leao Rodríguez-Fierros, Israel Pérez-Torres

**Affiliations:** 1 Research Direction Instituto Nacional de Cardiología Ignacio Chávez, Juan Badiano 1, Sección XVI, Tlalpan, Mexico City 14080, Mexico; elena.soto@cardiologia.org.mx; 2 Department of Cardiovascular Biomedicine, Instituto Nacional de Cardiología Ignacio Chávez, Mexico City 14080, Mexico; loe_mana@hotmail.com (L.M.-P.); medelhafidi@yahoo.com (M.E.-H.); 3 Department of Physiology, Instituto Nacional de Cardiología Ignacio Chávez, Juan Badiano 1, Sección XVI, Tlalpan, Mexico City 14080, Mexico; veronica.guarner@cardiologia.org.mx; 4 Critical Care Units of the Temporal COVID-19 Unit, Citibanamex Center, Mexico City 11200, Mexico; a2novi@hotmail.com (A.P.-C.); rrvaldezvazquez@gmail.com (R.R.V.-V.); 5 Departamento de Instrumentación Electromecánica, Instituto Nacional de Cardiología Ignacio Chávez, Mexico City 14080, Mexico; raulmmemije@yahoo.com; 6 Laboratorio de Patología Veterinaria, Facultad de Ciencias Naturales, Universidad Autónoma de Querétaro, Santiago de Querétaro 76230, Mexico; felixleao@hotmail.com

**Keywords:** SARS-CoV-2, COVID-19, vitamin E, fatty acids, phospholipids, prostaglandins

## Abstract

SARS-CoV-2 is an obligatory intracellular pathogen that requires a lipid bilayer membrane for its transport to build its nucleocapsid envelope and fuse with the host cell. The biological membranes are constituted by phospholipids (PLs), and vitamin E (Vit E) protects them from oxidative stress (OS). The aim of this study was to demonstrate if treatment with Vit E restores the modified profile of the FA in PLs in serum from patients with coronavirus disease-19 (COVID-19). We evaluated Vit E, total fatty acids (TFAs), fatty acids of the phospholipids (FAPLs), total phospholipids (TPLs), 8-isoprostane, thromboxane B_2_ (TXB_2_), prostaglandins (PGE_2_ and 6-keto-PGF1_α_), interleukin-6 (IL-6), and C-reactive protein (CRP) in serum from 22 COVID-19 patients before and after treatment with Vit E and compared the values with those from 23 healthy subjects (HSs). COVID-19 patients showed a decrease in Vit E, TPLs, FAPLs, and TFAs in serum in comparison to HSs (*p* ≤ 0.01), and Vit E treatment restored their levels (*p* ≤ 0.04). Likewise, there was an increase in IL-6 and CRP in COVID-19 patients in comparison with HSs (*p* ≤ 0.001), and treatment with Vit E decreased their levels (*p* ≤ 0.001). Treatment with Vit E as monotherapy can contribute to restoring the modified FA profile of the PLs in the SARS-CoV-2 infection, and this leads to a decrease in lipid peroxidation, OS, and the inflammatory process.

## 1. Introduction

The type 2 coronavirus that causes severe acute respiratory syndrome (SARS-CoV-2) is an obligatory intracellular pathogen that kidnaps the cell machinery that it needs for its replicative cycle. Lipidic interactions are essential during the entrance of this virus to the host cell and afterward for the budding process of new virions [1]. The first step of the viral infection is the union of the spike protein (S) of the virus with (subunit S1) the angiotensin-converting enzyme 2 (ACE2), which contributes to a conformational change in the S2 subunit of the S protein, resulting in the binding of the virus with the transmembrane protein serine protease 2 (TMPRSS2). Both the TMPRSS2 and ACE2 are present in the lipid rafts that are microdomains in the cell membrane of the host [2]. SARS-CoV-2 may use two mechanisms for its internalization: (1) fusion and (2) endocytosis. When fusion occurs, the viral glycoprotein S interacts with surface cell receptors, ACE2, as already mentioned, and then there is fusion of the membranes of the virus and the host. Endocytosis happens after fusion, and it is accompanied by invagination and transport. In this step, a diminished pH is required, which favors the union between the endosomal membrane and the viral envelope and the subsequent liberation of the nucleocapsid to the cytoplasm [2]. However, the degree of virulence also depends on the pH of the extracellular environment, the membrane rigidity, the density of the receptors, and the amount of the viral spike proteins [3].

Lipids including phospholipids (PLs), fatty acids (FA), and cholesterol (CT) are essential nutrients that play important roles in anatomical and physiological functions such as cell signaling, death, and survival pathways in all cells. Furthermore, they are components of the bilipid plasma membrane and the membranes of the organelles [4]. Bilipid membranes are conformed by CT and PL molecules, which constitute 50% of the total mass of the membrane, and the rest are constituted by structural proteins [4]. In turn, PLs are made up of two FAs and a phosphate molecule. SARS-CoV-2 can reprogram the metabolism of the FA in the host cell to obtain it for the synthesis of the PLs necessary for the formation of the viral membrane of new virions. In this sense, the isomers of the transcription factor sterol regulatory element-binding protein (SREBP) are involved [5]. The viral infection also stimulates the production of the bioactive FA mediators that participate in the host immune response, such as prostaglandins (PGs), thromboxanes (TXs), and leukotrienes (LKs), which are metabolites of the arachidonic acid (AA) pathway and are formed by cyclooxygenases (COXs), thromboxane synthase, lipo-oxygenase (LOX), and cytochrome P450 (CYP). These mediators participate as pro- and anti-inflammatory molecules in the infection process [1]. Other FAs that may also play a pivotal role in the replication of SARS-CoV-2 are palmitic (PA), oleic (OA), linoleic (LA), stearic, and dihomo-γ-linoleic (D-γ-LA) acids. These FAs are very important for the formation and function of the viral membrane [1,4]. In this sense, SARS-CoV-2 may contribute to an increase in the activity of the FA synthase (FAS) in the host cell. This enzyme is key in the synthesis of PA and is then used as a raw material for the synthesis of other FAs and becomes part of the lipid bilayer of membranes of the virus and facilitates virus propagation [6].

In addition to FAs, other lipids, such as CT, which are abundant in the lipid rafts of the membrane where the ACE2 receptor is anchored, are important in viral pathways, such as their biosynthesis and assembly [7]. Therefore, an intricate disturbance in lipid metabolism might happen in the process of infection and replication of SARS-CoV-2 for the benefit of the virus. Nevertheless, this leads to a decompensation of the pathways for lipid synthesis in the host that contributes to the clinical picture present in the patient, with a probable fatal outcome. The breakage of this intricate balance during the infection process is essential to reduce the clinical–pathological picture present in the patient. In this context, the regulation of lipid metabolism can be used as an adjuvant therapeutic target in patients infected by SARS-CoV-2 and as an addition to other strategies, including the battery of vaccines against this virus. In this sense, it has been demonstrated that supplementation with omega-3 FA (eicosapentaenoic acid EPA and docosahexaenoic DHA acid) or PLs enriched with EPA and DHA could reduce viral entrance to the host cells, ameliorate immune function, and diminishe the severity of some COVID-19 complications [8].

On the other hand, tocopherol, also known as vitamin E (Vit E), is an essential micronutrient and fat-soluble antioxidant in mammals that prevents the oxidation of FAs of the cell membranes by radical oxygen species (ROS), thus contributing to an increased fluidity of membranes and a decrease in lipid peroxidation (LPO) associated with the Haber–Weiss reaction [9]. The rate of tocopherol decay is α > β > γ > δ, in association with the biological potencies of these forms of Vit E [9]. Even though deficiencies in Vit E are rare in humans, they may occur by intestinal malabsorptive disorders, However, when they occur, they may lead to spinocerebellar ataxia, a decrease in motor control, lesions in both muscle and peripheral nerves, retinitis pigmentosa, macular degeneration, anemia, and lipoprotein disorders [10]. Vit E also compromises the deficiency of both cell-mediated and humoral immune functions in animal trials. Moreover, supplementation with Vit E strengthens immunity and increases resistance against several pathogens [11]. Moreover, Vit E acts synergistically with Vit C through the reduction in tocopheroxyl radicals [12].

Vit E supplementation (60 mg/7 day) decreased the LPO index in blood plasma and in the lung and liver in male mice with influenza [13]. However, only a limited number of human trials have shown the effects of Vit E on resistance against infectious diseases, such as sepsis, compared to those performed in animals [14]. The focus of these human trials was aimed at the elderly, who frequently contract respiratory diseases. Treatment with Vit E as a monotherapy has been poorly studied in COVID-19 despite the proposed beneficial effect of this treatment in various review manuscripts in the literature [15,16]. Moreover, adjuvant therapy with Vit E supplementation could decrease the production of ROS and restore the balance in the alteration of the metabolism of PLs in patients with COVID-19, thus contributing to a decrease in the clinical manifestation in the progression of the infection [17]. Therefore, the aim of this study was to demonstrate if treatment with Vit E as monotherapy is capable of restoring the modification of the FA in the PL profile in serum in patients with COVID-19.

## 2. Materialesand Methods

### 2.1. Ethical Considerations

Ethical approval was obtained on 19 August 2020 (Control-9867/2020, register REG. CONBIOETICA-09-CEI-01120160627). The protocol was registered (TRIAL REGISTRATION: ClinicalTrials.gov Identifier: NCT04570254). The Research and Ethics Committee of our institution approved the research protocol (Institutional protocol number: 22-1289). This study was carried out according to the international ethical standards and the General Health Law, as well as  the Helsinki Declaration, modified at the Congress of Tokyo, Japan. An informed consent form written for recruitment and the use of patient data were obtained from each patient, control subject, or their legal representative

### 2.2. Population of this Study

This was a prospective, analytical, open, and longitudinal (before–after) study run on 22 patients with COVID-19 who received Vit E treatment. The group before treatment with Vit E was compared with 23 healthy subjects (HSs), who constitute the control group. Inclusion criteria were patients with SARS-CoV-2 infection, who were 18 years old or older, belonged to both sexes with or without comorbidities, admitted to the intensive care unit (ICU) of the CITIBANAMEX Center, developed or did not develop septic shock and secondary to moderate or severe pneumonia by SARS-CoV-2 infection, and able to grant informed consent. The COVID-19 patients were considered to have septic shock when there was an acute increase in at least 2 points in the Sequential Organ Failure Assessment (SOFA) score, which includes the stage of neurological, respiratory, hemodynamic, hepatic, and hematologic conditions; they had lactate levels ≥ 2 mmol/L, and according to the conditions, the patients were dependent on a vasopressor for at least 2 h before recruitment. The SOFA score was also evaluated at admission and during the days of the treatment to determine organ dysfunction. Exclusion criteria were patients that were under chronic use (last 6 months) or recent use of Vit E and antioxidants, such as Vit C, statins, and steroids, patients that were not able to grant informed consent or refused to be included, and pregnant women or women that were breastfeeding.

**Figure 1:**
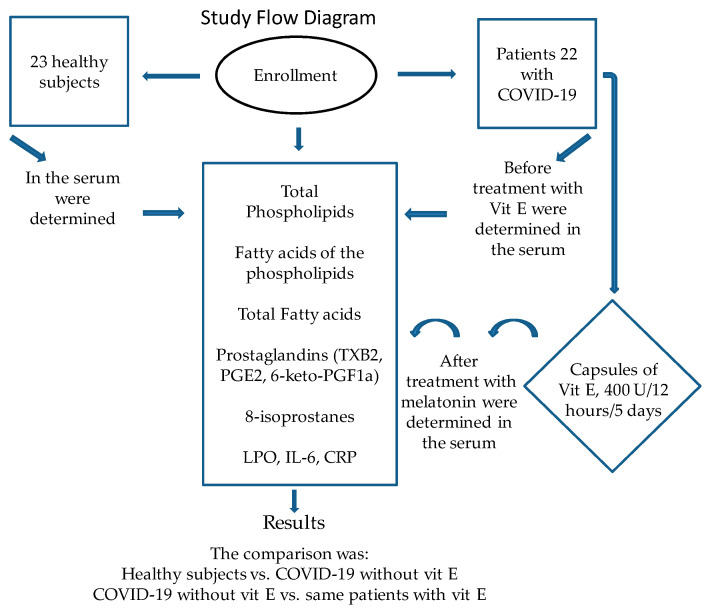
Flow diagram used during this study.

Treatment with Vit E was applied during the 2020 pandemic between the months of August and September [18] and consisted of Vit E (α-tocopheryl acetate) capsules of 400 IU equivalent to 800 mg that were administered every 12 h/5 days. The patients were not vaccinated against SARS-CoV-2 because a vaccine had not yet been approved at the time this study was carried out. Hospitalized patients with COVID-19 were classified as severe or moderate according to their ventilatory status. COVID-19 patients with the severe condition require invasive mechanical intubation according to the Berlin criteria for acute respiratory distress syndrome (ARDS) [19]. Patients were given individualized management according to an algorithm suggested by Soto et al., and they were not given hydroxychloroquine or antivirals [20]. Treatment began before the recognition of the presence or absence of septic shock and during the first hour after admission. Depending on the hemodynamic status, management with crystalloid solutions and/or albumin was taken into account by means of dynamic indicators. If necessary, vasopressors were given to keep a mean arterial pressure (MAP) ≥ 65 mmHg. Inotropic drugs (dobutamine) were administered when myocardial dysfunction was present. Norepinephrine (NE) was the first option and/or vasopressin was used when there was a need to increase the MAP or reduce the NE dose. When there was a decrease in hemoglobin (<7.0 g/dL) in the absence of severe hypoxemia, myocardial ischemia, or severe bleeding, transfusion of blood packs was used. Mechanical ventilation with initial volumes of 6 mL/kg was used in ARDS patients [19]. Plateau pressure was maintained at ≤30 cm H_2_O, and alveolar conduction pressure was ≤13 cm H_2_O. Positive end expiration pressure titration was managed by the use of the fraction of the inspired oxygen/positive end expiration pressure (FiO_2_/PEEP). Treatment with anticoagulants was based on the Thatched guidelines [21]. Management with the prone position was necessary in patients with PaO_2_/FiO_2_ of ≤150 mmHg [22]. Standard therapeutic management with dexamethasone 8 mg, i.v., every 24 h for 7 days in all patients was given between 1 and 21 days of the onset of symptoms when not counterindicated. Pentoxifylline tablets of 400 mg were applied every 12 h by an oral route or a nasal-enteral tube for 5 days [18]. They were counterindicated when there was a requirement of O_2_ > 3 L, a progressive requirement of =2, PaO_2_/FiO_2_ ≤ 250 mmHg, O_2_ use plus bilateral infiltrates in the radiography, O_2_ use plus DHL ≤ 250 U/L or ferritin ≥ 300 or DD ≥ 1000 ng/mL, or CPK ≥ 2 times the upper normal value. The following conditions were not considered as counterindications or relative counterindications: glucose > 250 mg/dL with hypoglycemia, hypokalemia < 3.3 meq, blood pressure > 155/95 mmHg with antihypertensive treatment, glaucoma, triglycerides (TGs) > 500 mg/dL (start treatment), a history of known peptic ulcers or bleeding from recent gastrointestinal tracts, untreated or decompensated dementia or psychiatric illness, and the use of non-potassium sparing diuretics or the use of inhaled B_2_ agonists. The next conditions were monitored at follow-up: pre-prandial capillary glucose (7–13–1 h) for 10 days, even in fasting patients, MAP per shift, and basal potassium every 72 h. Some of the results related to this study were previously reported by Chavarría et al. during the before and after treatment with Vit E treatment evaluation [18]. Figure 1 shows a flowchart of the recruitment of the patients and treatment with Vit E in the patients with COVID-19.

### 2.3. Healthy Subjects

A total of 15 men and 8 women matched by age and gender who were negative for SARS-CoV-2 infection were included in the group of healthy subjects (HSs) and served as the controls. Autoimmune diseases, thyroid disease, obesity, overweight, dyslipidemia, arterial hypertension, diabetes mellitus, and inflammatory and degenerative diseases were not present in the HSs. The intake of antioxidant and non-steroidal anti-inflammatory drugs in HSs that could interfere with the results of this study was suspended 48 h before the sample was obtained. Biochemical variables such as glucose, calcium (Ca^2+^), creatinine, uric acid, CT, TGs, high-density lipoproteins (HDLs), low-density lipoproteins (LDLs), and C-reactive proteins (CRPs) were determined.

### 2.4. Detection of SARS-CoV-2 by a Real-Time Reverse Transcriptase Polymerase Chain Reaction

Swab samples were collected from COVID-19 patients to apply the Paired Technique (nasopharyngeal and saliva). Samples were considered positive for SARS-CoV-2 when both the N1 and N2 protein primer presets were detected. Specific probes to detect the virus and the real-time reverse transcriptase polymerase chain reaction technique (qRT-PCR) were employed to determine the presence of the SARS-CoV-2 virus.

### 2.5. Peripheral Blood Samples

A total of 10 mL of peripheral blood per patient were collected by venopuncture. The blood was centrifuged for 20 min at 936 g and 4 °C. The red blood cell pellet was discarded; the serum was recovered and collected in polypropylene tubes of 400 μL and stored at −30 °C until use. Interleukin-6 (IL-6), CRPs, TGs, HDLs, LDLs, glucose, urea nitrogen, creatinine, CT, Ca^2+^, hemoglobin, leukocytes, lymphocytes, platelets, albumin, D-dimer, fibrinogen, and ferritin were determined in the serum from the COVID-19 patients. Data from the patient’s medical history, including demographic data, prior illnesses to SARS-CoV-2 infection, COVID-19 test results, whether mechanical ventilation was used, and the type of treatment given, were used for the analysis of the results.

### 2.6. Determination of Markers of Lipid Peroxidation

The LPO index was measured indirectly through malondialdehyde MDA, and it was measured with a UV-2100 spectrophotometer at 532 nm (United Products and Instruments, Inc., 182-E Ridge Road Dayton, NJ, USA). A total of 100 μL of serum were used for this determination. Methanol with BHT at 4% (100 μL) and a KH_2_PO_4_ buffer pH 7.4 (1 mL) were added to the sample and incubated at 37 °C for 30 min. Then, 2-thiobarbituric acid at 0.8 M (1.5 mL) was added and incubated at 90 °C for 1 h. Afterward, KCl at 5% (1 mL) plus n-butanol (4 mL) were added, and the sample was shaken for 30 sec and centrifuged at 4000 rpm for 2 min. The organic phase was extracted, and the absorbance was measured.

### 2.7. 8-Isoprostane, TXB_2_, PGE_2_, 6-keto-PGF_1α_, and IL-6 Determinations

A total of 50 μL of serum were used for these determinations. The quantification of 8-isoprostane (#516351 ELISA Kit), thromboxane B_2_ (TXB_2_) (#501020 ELISA Kit), PGE_2_ (#514531 ELISA Kit), and 6-keto-PGF_1α_ (#515211 ELISA Kit) were provided by Cayman chemical (1180 E. Ellsworth Rd., Ann Arbor, MI, USA), and IL-6 (#D6050B) was provided by R&D systems as a biotechne and was measured at a wavelength range of 450–492 nm using a visible light microplate reader (Stat Fax 3200 Awareness Technology, Palm City, FL, USA), according at the manufacturer’s specifications.

### 2.8. Vitamin E Quantification in Serum

For the extraction and derivatization of Vit E, saline solution 0.09% (1 mL respectively) and hexane (3 mL) were added to 100 µL of serum diethyl ether, and the samples were shaken with a vortex for 30 s and centrifuged at 3000 rpm for 4 min. The organic phase was recovered and evaporated under a stream of nitrogen. The residue was suspended with pyridine (200 mL) and hexamethyldesilazane (100 mL) and shaken in a vortex for 30 s. Then, chlorotrimethylsilane (50 mL) was added and derivatized at 60 °C for 20 min, and then hexane (3 mL) was added. The organic phase was recovered, filtered, and evaporated with a gentle stream of nitrogen [23]. The calibration curve was carried out with 400 U α- tocopherol as standard and 12.5, 25, 50, 100, and 200 μL corresponding to 0.04, 0.08, 0.16, and 0.32 activity units. Vit E was identified by gas chromatography FID in a Carlo Erba Fratovap 2300 chromatograph (Calle Filadors, Sabadell, España) equipped with a capillary column packed with the stationary phase HP-FFAP (description: 30 m length × 0.320 mm diameter × 0.25 µm film) and fitted with a flame ionization detector at 240 °C with helium as the carrier gas at a flow rate of 1.2 mL/min. The areas under the peak were calculated using Cromatograf software version 1.1 coupled to a gas chromatograph.

### 2.9. Total Fatty Acid (TFA) Determination

For the extraction and derivatization of the TFA, 100 µL of serum were used according to the method described by Folch et al. [24] in the presence of 50 µg of margaric acid (C17:0) as an internal standard. Then, 1 mL of a saline solution (0.09%) was added and mixed for 15 s, and then 2 mL of a methanol chloroform mixture (2:1 vol/vol) plus 0.002% BHT were added and centrifuged at 3000 rpm by 5 min. This step was repeated twice, and the organic phase was recovered and evaporated under a gentle current of nitrogen (N_2_). TFAs were transesterified to their FA methyl esters by heating them at 90 °C for 2 h with 2 mL of methanol plus 0.002% BHT, 40 µL of H_2_SO_4_, and 100 µL of toluene. Afterward, 1 mL of the saline solution and 4 mL the hexane were added, and the mixture was centrifuged at 3000 rpm for 5 min. The hexane phase was recovered and evaporated under a gentle current of N_2_. The evaporated residue containing the FA was suspended in 100 µL of hexane, and 4 µL was injected into the chromatograph. The TFA methyl esters were separated and identified by gas chromatography FID in a Carlo Erba Fratovap 2300 chromatograph equipped with a capillary column packed with the stationary phase HP-FFAP (description: 30 m length × 0.320 mm diameter × 0.25 µm film) and fitted with a flame ionization detector at 210 °C with helium as the carrier gas at a flow rate of 1.2 mL/min. The areas under the peaks were calculated using Cromatograf software version 1.1 coupled to the gas chromatograph. The identification of each FA methyl ester was made by comparing their retention time with their corresponding standard [1].

### 2.10. Fatty Acid Analysis in an Acetone Insoluble Phospholipid Fraction (FAAIPF) Determination in Serum

For fatty acid analysis in an acetone insoluble phospholipid fraction (FAAIPF) extraction, 200 μL of serum were used in the presence of 50 μg of L-γ-phosphatidylcholine-di-heptadecanoyl acid as internal standard plus acetone (1 mL) and 1 mL of saline solution (0.09%). The mixture was shaken 30 s and centrifuged at 1145 g at room temperature for 4 min. The supernatant was removed, and the button was suspended with 1 mL of the saline solution (0.09%) and mixed for 15 s, Then, a mixture of the chloroform–methanol with BHT (0.002%) was added (2:1, vol/vol, 4 mL) according to the method described previously [24]. FAAIPFs were transesterified to their FAPL methyl esters and separated as described above [1]. The acetone-insoluble phospholipid fraction is phosphatidyl choline, phosphatidyl ethanolamine, phosphatidyl inositol, and phosphatidic acid [25].

### 2.11. Total Phospholipids (TPLs)

The TPL determination in the serum was made with 100 µL of serum by a colorimetric assay through an enzymatic method utilizing N-ethyl-N-(2,hydroxy-3-sulfopropyl)-3,5-domethoxyaniline, according to the manufacturer’s recommendations (Fujifilm (Phospholipids C, Code No. 997-01801, FUJIFLIM Wako Diagnostic U.S.A. Corporation, 1600 Bellwood Road, Richmond, VA, USA). The absorbance was measured at 600 nm. This method is able to detect lecithin, lysolecithin, and the sphingomyelin of phospholipids, but phosphatidyl ethanolamine is not measured in the sample.

### 2.12. Statistical Analysis

TFAs and PLs are expressed as a percentage. Categorical variables were expressed as frequencies and percentages. Continuous variables were compared with the Mann–Whitney U rank-sum test followed by the normality test (Shapiro–Wilk) between HSs vs. before treatment with Vit E in the COVID-19 patients and by the Kruskall–Wallis test before vs. after treatment with Vit E in the COVID-19 patients. The sample size was calculated by a paired test of two correlated means, specifying the standard error of the differences. The calculation was taken according to the data found in our study and previously reported in the article by Chavarría et al. [18]. The calculation was based on two forms. The first was the percentage of cases with elevated LPO pre-treatment and post-treatment, which ranged between 1.75 and 1.05 with an estimated delta of 0.80 and an α error of 0.05 and 0.01, as well as a power of 0.84 and 0.99, respectively. We decided to take the calculation with an alpha error of 0.05 and a power of 84, and we included 22 patients, even if only 13 were required. Sigma Plot^®^ version 15 (Systat Software Inc., SanJose, CA 95131, USA, EE.UU, North First Street, Suite 360, Jandel Corporation, San Jose, CA, USA) was used to generate the analysis and graphs. Differences were considered statistically significant when *p* ≤ 0.05.

## 3. Results

### 3.1. Demographic Characteristics of the Patients with COVID-19

A total of nineteen men (86%) and three (14%) women were included in the group of patients, with a median age of 55 years and a body mass index of 28. The comorbidities they presented were diabetes mellitus (27%), hypertension (14%), dyslipidemia (46%), overweight (41%), and morbid obesity (36%). Furthermore, the gasometry, blood biochemistry, and organ failure sequential score, along with demographic characteristics, are shown in Table 1. The values are expressed as medians with a minimum and maximum range, and some values are reported as percentages.

### 3.2. Blood Biochemical Characteristics of the Healthy Subjects and COVID-19 Patients

A total of fifteen men (55%) and eight (35%) women were included in the HS group, with a median age of 60 years, adding to a total of 23 individuals. The values of the levels of glucose, Ca^2+^, uric acid, creatinine, CT, HDLs, LDLs, TGs, and CRPs in these subjects are shown in Table 2. The same variables in the serum of the patients with COVID-19 were also analyzed and are also shown in Table 2. The COVID-19 patients showed a decrease in glucose levels, creatinine, and CT LDL (*p* ≤ 0.01) and an increase in CRP (0.01) in comparison with the HSs.

Table 3 shows the values of IL-6 and CRP in the HS and COVID-19 patients before and after treatment with Vit E. COVID-19 patients before treatment with Vit E showed an increase in these pro-inflammatory markers in comparison with HSs (*p* = 0.001), but a decrease in both variables (*p* = 0.01) was observed after treatment with Vit E.

### 3.3. Vit E and Total Phospholipids

**Figure 2:**
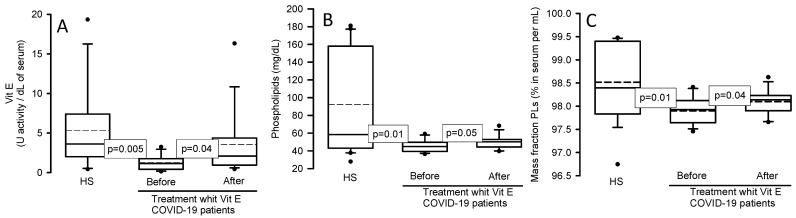
Vit E levels, TPLs, and the mass fraction of the PLs in the serum of COVID-19 patients and HSs before and after treatment with Vit E. Vit E, TPLs, and the mass fraction of the PLs showed a significant decrease in COVID-19 patients when compared to HSs, but after treatment with Vit E, the levels were restored. Panel (**A**), Vit E; panel (**B**), TPLs; and panel (**C**), the mass fraction of the PLs. The dark circles that stand out from each bar are the outliers. The results are shown as the median, first quartile, third quartile, and half dotted line. Abbreviations: Vit = vitamin, TPLs = total phospholipids.

Figure 2A shows that the Vit E concentration in COVID-19 patients was decreased (*p* = 0.005) in comparison to HSs. However, treatment with Vit E favored an increase in these patients (*p* = 0.04). Figure 2B shows that the TPLs determined by colorimetry, which are represented by lecithin, lysolecithin, and the sphingomyelin of phospholipids, were decreased in the COVID-19 patients (*p* = 0.01) in comparison with HSs and that treatment with Vit E increase them (*p* = 0.05). Figure 2C shows the mass of the fraction of the PLs in the serum, where there was a significant decrease (*p* = 0.01) in COVID-19 patients versus HSs, and that treatment with Vit E favored a significant increase (*p* = 0.04).

### 3.4. Total Fatty Acids and Fatty Acids in an Acetone Insoluble Phospholipid Fraction (FAAIPF) in Serum

Table 4 shows the percentage of TFAs in the serum of the HSs and patients with COVID-19. The PA, palmitoleic, and stearic, (*p* ≤ 0.04) acids were decreased, but OA was increased (*p* = 0.001) in patients with COVID-19 in comparison to HSs. The AA and EPA showed a tendency to increase but did not reaching a significant difference. However, treatment with Vit E increased the stearic, γ-linoleic, α-linoleic, D-γ-LA, and DHA (*p* ≤ 0.02). The EPA showed a tendency to increase but did not reaching a significant difference. The same table also shows the TFA expressed in mg per mL in the serum, where treatment with Vit E only presented a tendency to decrease (*p* = 0.07) without there being significant changes between the HS and COVID-19 patients.

Table 5 shows the composition of the saturated (SFA), monounsaturated (MUFA), and polyunsaturated-3, -6 (PUFA) acids in the serum in the patients with COVID-19 before and after treatment with Vit E and in the serum in the HSs. The total SFA decreased (*p* = 0.02) and the MUFA increased (*p* = 0.02) in patients with COVID-19. However, treatment with Vit E favored an increase in the PUFA-6 (*p* = 0.01).

Table 6 shows the desaturation indexes, including the palmitoleic/PA ratio, which was decreased (*p* = 0.01), and the stearic/OA, ratio which was increased (*p* = 0.001). These ratios are usually used as indexes of ∆^9^—the activity of desaturation. The ratio of γ-linoleic/linoleic that is used as an index of the desaturation ∆^6^ increased (*p* = 0.001) in COVID-19 patients in comparison to HSs. However, treatment with Vit E in COVID-19 patients decreased (*p* = 0.01). The AA/D-γ-LA index was increased (*p* = 0.001), and the DHA/EPA tended to increase but did not reaching a significant difference (*p* = 0.07). These indexes are used as indexes of ∆^5^—the desaturase activity.

Table 7 shows the FAAIPF percentage in the serum from the HSs and patients with COVID-19. The PA and stearic (*p* = 0.002 and *p* = 0.01, respectively) increased, but OA and D-γ-LA tended to increase (*p* = 0.07), and LA acid decreased, (*p* = 0.001) in patients with COVID-19 in comparison to HSs. But, treatment with Vit E decreased the PA and D-γ-LA (*p* = 0.03 and *p* = 0.01), respectively. The EPA and DHA showed a significant increase (*p* = 0.01).

Table 8 shows the composition of the SFA, MUFA, and PUFA-3 and -6 in the FAAIPF in serum in the patients with COVID-19 before and after treatment with Vit E and in the HSs. The total SFAs increased (*p* = 0.002), but the PUFA-3 decreased (*p* = 0.002). However, treatment with Vit E favored an increase in the PUFA-6 (*p* = 0.03) and tended to decrease the SFA (*p* = 0.07).

Table 9 shows the desaturation index in the FAAIPF. The stearic/OA ratio decreased (*p* = 0.03) in COVID-19 patients in comparison to HSs, but treatment with Vit E in the COVID-19 patients tended to increase the index of the ∆^5^ desaturase through the DHA/EPA ratio, but there was no significant difference (*p* = 0.07).

### 3.5. Prostaglandins and Interleukin Concentration

**Figure 3:**
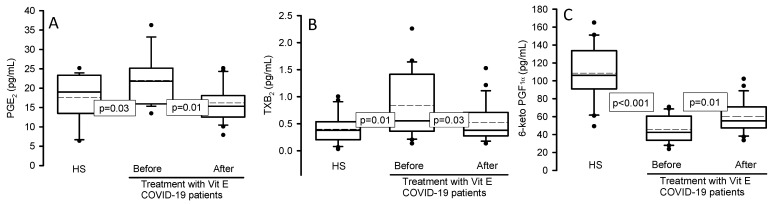
Prostaglandin concentrations in the serum of the HSs and COVID-19 patients before and after treatment with Vit E. PGE_2_ (panel **A**), TXB_2_ (panel **B**), and 6-keto PGF_1α_ (panel **C**). PGE_2_ and TXB_2_ showed a significant increase in COVID-19 patients compared with the HSs, but after treatment with Vit E, a decrease was observed. However, the contrary effect was present in the 6-keto PGF_1α_ concentration. The dark circles that stand out from each bar are the outliers. The results are shown as the median, first quartile, third quartile, and half dotted line. Abbreviations: Vit = vitamin, HS = healthy subject, PGE_2_ = prostaglandin E_2_, TXB_2_ = thromboxane B_2_, 6-keto PGF_1α_ = 6-keto prostaglandin F 1 alpha.

Figure 3A shows the concentration of PGE_2_ in the serum of the experimental groups; there was a significant increase in patients with COVID-19 in comparison with HSs (*p* = 0.03), and after treatment with Vit E (*p* = 0.01), it decreased. The same tendency was observed with the TXB_2_ concentration in the patients with COVID-19 (Figure 3B) in comparison to HSs (*p* = 0.01) and after treatment with Vit E (*p* = 0.03). However, 6-keto PGF_1α_ decreased in the patients with COVID-19 vs. the same patients after treatment with Vit E in HSs (*p* < 0.001 and *p* = 0.01 respectively, Figure 3C).

### 3.6. Lipid Peroxidation and 8-Isoprostene

**Figure 4:**
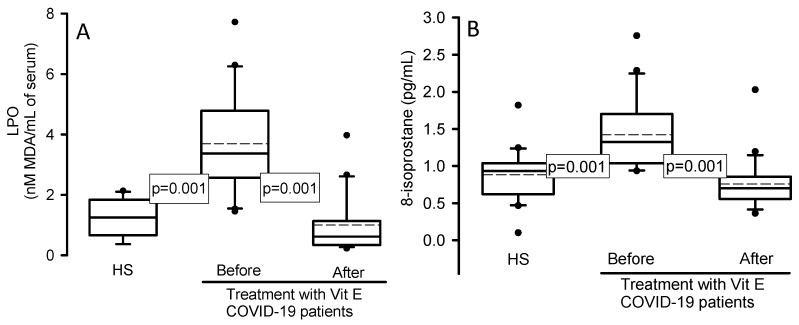
LPO (panel **A**) and 8-isoprostane (panel **B**) concentration in the serum in HSs and COVID-19 patients before and after treatment with Vit E. LPO and 8-isoprostane showed a significant increase in COVID-19 patients compared with HSs, but after treatment with Vit E, they decreased. The dark circles that stand out from each bar are the outliers. The results are shown as the median, first quartile, third quartile, and half dotted line. Abbreviations: Vit = vitamin, HS = healthy subject, LPO = lipid peroxidation.

Figure 4A shows that the LPO index and 8-isoprostane (Figure 4B) were significantly increased in the serum of the COVID-19 patients in comparison with HSs (*p* = 0.001) after treatment with Vit E (*p* = 0.001).

## 4. Discussion

The SARS-CoV-2 virus responsible for the COVID-19 pandemic that devastated the world between 2020 and 2021 continues to mutate and still claims the lives of millions of people around the world. Although there are already different vaccines provided by different pharmaceutical companies against this virus (13.590 million vaccine doses have been administered and complete vaccination charts have been given to around 37% of the population, and the total population vaccinated with at least one booster dose of a COVID-19 vaccine is 32%), recent data from the WHO Coronavirus (COVID-19) Dashboard show that 7,044,637 people have died worldwide. For this reason, it is of vital importance to continue understanding both the metabolic pathways compromised by this virus in the patients and the possible use of adjuvant therapeutic agents that may contribute to combatting it. Adjuvant agents lead to lesser manifestations of the clinical data in patients and help to reduce the number of deaths. In this sense, Vit E, at a dose of 400 IU/day, has been utilized as an important anti-inflammatory and anti-OS therapy in humans with immunodeficiency and has a positive influence on the presence of the virus with promising results. This treatment decreases the viral load [26]. In addition, it has been demonstrated that a decrease in Vit E is present in the plasma of patients with COVID-19 [27]. Another study showed that in 49 patients diagnosed with COVID-19, a Vit E deficiency of 7% was present [28]. Moreover, Vit E levels were lower in pregnant women with COVID-19 [29]. Patients with COVID-19 also show an alteration in lipid metabolism characterized in part by a decrease in plasma PLs [30], which has been associated with Vit E deficiency. In this sense, the aim of this study was to demonstrate if treatment with Vit E restores the modification of the profile of the FA in the PLs in serum in patients with COVID-19.

Our results show a decrease in Vit E levels in the serum of patients with COVID-19, which confirms the previously mentioned reports [27] and suggests that in the infectious process caused by SARS-CoV-2, there is a depletion of molecules with antioxidant capacity, such as Vit E, which contributes to the progression of the infection and is associated with the inflammatory degree [31]. In this sense, IL-6 and CRP, which are pro-inflammatory molecules, were elevated in the serum of the COVID-19 patients in our study. The rising viral load in COVID-19 results in a fast increase in inflammatory monocytes and neutrophils in the lungs in an effort to try to counteract the infection. These molecules further activate ILs and PGs that continuously and irreversibly affect the lung tissue [32]. However, treatment with Vit E decreased the IL-6, CRP, PGE_2_, and TXB_2_ levels and increased 6-keto-PGF_1α_, which is an anti-inflammatory molecule. These results suggest that it is necessary to maintain and restore the levels of Vit E in the serum during the infection to reduce or control the progression of inflammation. A previous study evaluated the association between Vit E and COVID-19 and hypothesized that Vit E could amplify the immune system due to its antioxidant properties and its roles in maintaining the integrity of the T-cell membranes, thus reducing the duration of the infection [33]. Therefore, our results suggest that treatment with Vit E effectively regulates the immune activity that confers protection against SARS-CoV-2 by decreasing IL-6 and CRP [34]. In this regard, it has been reported that the most important effects of Vit E supplementation on immune system activities include post-mitogenic stimulation, lymphocyte proliferation, and an increase in delayed-type hypersensitivity [35]. Studies in vitro and in vivo have demonstrated that Vit E treatment improves naive T-lymphocytes, natural-killers, and dendritic cell activities to promote the initiation of T-cell activation. It also inhibits the production of pro-inflammatory cytokines, such as IL-1, -6, -8, and TNF-α, but stimulates the IFN system, which exerts antiviral activities [35].

Moreover, high levels of LPO are present in the inflammatory process, and these are in part the result of ferroptosis present in these patients [36]. Our results showed an increase in the LPO index in patients with COVID-19 in comparison to HSs. Moreover, treatment with Vit E favored a decrease. This suggests that Vit E supplementation is capable of decreasing the LPO index associated with the increase in ferroptosis [37]. In this regard, Vit E reacts with peroxyl radicals to prevent the formation of lipid hydroperoxides associated with the FA oxidation of the cell membrane. Afterward, glutathione (GSH) and enzymes that employ GSH may detoxify oxidized lipids [38]. Furthermore, Vit E deficiency is related to ferroptosis, and other studies suggest that Vit E supplementation at a high dose of 500 mg/kg may act as a treatment to inhibit ferroptosis in COVID-19 patients and decrease the damages caused by ferroptosis to multiple organs, such as the lung, kidney, liver, gut, heart, and nervous system [39]. Our results show an increase in ferroptosis in COVID-19 before treatment with Vit E. However, we could not validate whether ferroptosis decreased in these patients after treatment with Vit E, and, therefore, more studies are required to confirm this. Nevertheless, there was a significant decrease in LPO.

The increase in LPO in COVID-19 suggests that an oxidizing background in the SARS-CoV-2 infection has already been documented [38]. This situation favors the oxidation of the AA to 8-isoprostanes, which are molecules considered markers of LPO and oxidative injury. For this reason, this molecule has been considered an ideal marker in the broncho-alveolar lavage fluid of patients with interstitial lung disease [40]. Our results revealed a statistically significant elevation of 8-isoprostane in COVID-19 patients, and there was a decrease in its level after of treatment Vit E. This suggests that 8-isoprostane may be a predictive marker against the OS present in these patients and reinforces that Vit E supplementation contributes to decreasing the oxidizing background. In patients co-infected with malaria and COVID-19, treatment with Vit E decreases 8-isoprostane levels [41].

Regarding the results of the TPL fractions obtained (lecithin, lysolecithin, and sphingomyelin), the patients with COVID-19 showed a significant decrease in serum. Different studies in COVID-19 have demonstrated an alteration in the profile of the lipid metabolism in this disease [1,4,8]. Therefore, the rescheduling of the FA metabolism in the host seems to be essential [42]. The decrease in the mass fraction in the TPLs of the lecithin, lysolecithin, and sphingomyelin quantified could be due in part to their use in the formation of new viral membranes and the reparation of the membrane of the host cell after the budding process; they can also restore the high oxidation by free radicals that result in oxidized PLs, which contribute to the LPO index. However, treatment with Vit E was capable of diminishing this [43], and as the results demonstrate, the administration of 500 mg/d of Vit E for 12 weeks in patients with hepatitis C virus showed an increase in total PUFAs in red blood cells of the PLs [44] since it has been described that Vit E can exert its maximum protective effect in a concentration of 1 molecule for every 2000 PL molecules [45]. In addition, the analysis of the FAAIPF showed an increase in the PA and stearic acid and a decrease in the LA in this study. However, in the TFA, the PA, palmitoleic, and stearic acids showed a decrease. These results might seem paradoxical; however, these alterations may be due in part to the need for host membranes to be repaired as the budding process of new virions occurs. In this sense, our results show that the TFA in the serum tends to increase in COVID-19 patients. At this stage, there is a greater demand for the synthesis of FAs. There is also a great need for these molecules for the formation of the viral membrane and because they are necessary for binding to the receptor-binding domain, such as the LA [46]. However, treatment with Vit E may restore these requirements [47]. In this sense, it has been demonstrated that the FA of the PLs in the serum reflects the FA from PLs in the membranes of erythrocytes [48].

In addition, the desaturation process constitutes an important step in the metabolism of FAs, and desaturases are key enzymes in the biosynthesis of the MUFA from the SFA. These enzymes introduce a cis-double bond into the SFA, which is very important in the control of the structural, fluidity, and disorders of the membrane [49]. Our results show significant changes in the activities of ∆^9−^, ∆^6−^, and ∆^5−^ desaturases in the serum of the patients with COVID-19, but treatment with Vit E restored the activities of these enzymes, both in the FA and TPL. These results suggest that there is an alteration in the activity of desaturases in the SARS-CoV-2 infection, but supplementation with Vit E restores them. The disorders associated with an imbalance in the activity of desaturases include modifications in membrane ionic transport, receptor accessibility, and cellular enzymatic activities [50]. Our results evidenced the modification in the proportions of the SFA, MUFA, TFA, SFA, and PUFA n-3 of the FAAIPF. However, treatment with Vit E restored them and even increased PUFA n-6 in both the TFA and FAAIPF. This result suggests that there exists an alteration in the proportion of the SFA and MUFA in the SARS-CoV-2 infection but that the supplementation with Vit E restores it. The increase in PUFA n-6 or the tendency in PUFA n-3 in both the TFA and FAAIPF is important because even though we did not provide a diet with -3 or -6, these molecules were increased. These FAs influence the immune response. They are essential elements since there is a need to consume them in the diet because they cannot be endogenously synthesized. The PUFA-3 fatty acids largely comprise α-linolenic acid, DHAs, and EPAs. These FAs may trigger anti-inflammatory reactions in the body. Also, both PUFA-3 and -6 can change the composition of the PL bilayer of the host cell membrane, thereby preventing viral entry. When incorporated into the plasma membrane, they can affect the clumping of toll-like receptors associated with the prevention of signals that activate NF-κB, the production of other pro-inflammatory mediators, and the reduction in clinical complications in COVID-19 patients [51]. Furthermore, DHAs and EPAs also serve as precursors of resolvins, which reduce the production of pro-inflammatory mediators, eventually resulting in a decrease in systemic inflammation [52]. In this sense, there exists an interrelation between Vit E and PUFA n-3 and -6. For example, the supplementation with Vit E increased the levels of PUFA n-3 and decreased the levels of SFAs in hypertriglyceridemic rats [53]. In zebrafish, a deficiency of Vit E leads to a depletion of PUFAs [54], and the amount of Vit E present in tissues is related to the PUFA concentration, especially in the membranes [55].

**Figure 5:**
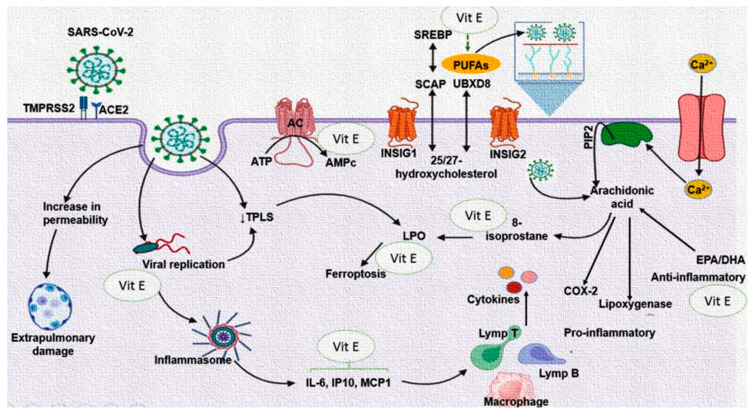
A decrease in Vit E and an alteration in the PLs of the plasma of the patients with COVID-19 has been observed and is associated with the increase IL-6, CRP, PGE_2_, TXB_2_, LPO index, ferroptosis, and 8-isoprotane. However, Vit E supplementation may decrease IL-6, CRP, PGE_2_, LPO, ferroptosis, TXB_2_, and 8-isoprotane levels and increase 6-keto-PGF_1α_. This could contribute to amplifying the immune system and decreasing the viral load. The treatment can also decrease the oxidation of PLs. These are used in the formation of new viral membranes and the reparation of the membrane of the host cell after the budding process, and this effect is attributed to Vit E, which can exert its maximum protective effect on approximately 2000 PL molecules [45].

Furthermore, the increase in the PUFA could contribute to a decrease in PGE_2_, TXB_2_ and favor a 6-keto-PGF1_α_ increase, which is observed in our results, and this may in part decrease the inflammatory state and thrombosis in the patients with COVID-19. Thus, Vit E may interact with different enzymes, such as protein kinases, protein phosphatases, lipid kinases, lipid phosphatases, lipid metabolic enzymes, and enzymes, involved in cyclic adenosine monophosphate metabolism [56]. It regulates a wide spectrum of key cellular processes, including many enzymes that are involved in pro-inflammatory events like COX_2_, phospholipase A_2_, and 5-, 12-, and 15-LOX [57]. Figure 5 summarizes the beneficial effect of treatment with Vit E on the reduction in the FA in the PL fraction oxidized in SARS-CoV-2 infection.

On the other hand, Vit E deficiency is related to a greater synthesis of the platelet-activating factor (PAF), which is produced by perivascular mast cells. The increase in the PAF leads to inflammation and high immunothrombosis in COVID-19 patients [58]. This has been associated with the antiphospholipid antibody syndrome (aPLs), which is an autoimmune thrombophilia mediated by autoantibodies directed against plasma phospholipid-binding proteins, mainly β2 glycoprotein I and prothrombin [59]. The exact mechanism by which these aPLs induce thrombosis is still not well understood in SARS-CoV-2 infection. In this sense, the results in different studies are controversial; for example, some papers report an increase while others a decrease [60,61]. However, there is evidence of an increase in aPLs antibodies in SARS-CoV-2 infection [62]. In this regard, a study that measured the concentration of aCL IgG antibodies in the positive serum of COVID-19 before and after vaccination with different vaccines or following SARS-CoV-2 infection was not clinically pathogenic for the risk of thrombosis [63]. However, another study demonstrated that vaccination did not trigger early autoantibody production [64]. Despite the above, treatment with Vit E has shown that it may inhibit the synthesis of the PAF, which induces platelet aggregation [65]. Although the relationship between aPLs and SARS-CoV-2 infection is not solid, a healthy diet containing PAF inhibitors, such as Vit E and flavonoids, could have an effect not only on inflammation but also the reduction in OS, thus preventing the harmful effects of thrombosis [66].

## 5. Conclusions

In conclusion, these results suggest that treatment with Vit E as monotherapy can contribute to restoring the FA profile of the PLs, which is modified by the SARS-CoV-2 infection, and this leads to a decrease in LPO, OS, and the inflammatory process. Therefore, treatment with Vit E could be used as an adjuvant therapy for a SARS-CoV-2 infection.

### 5.1. Perspectives

Although there are currently different vaccines against SARS-CoV-2 that allow us to reduce the severity of the infection, the administration of Vit E as an adjuvant therapy can decrease the oxidation of PLs in the cell membrane and contribute to increasing the innate immune response. Treatment with Vit E could also restore and decrease the intricate disturbance in the metabolism of lipids, which is an important step in the process of infection and replication of SARS-CoV-2.

### 5.2. Study Limitations

A limitation of this study is the small group of patients with COVID-19 that were included. Another limitation of this study is that the analysis of the FA of the FAAIPFs for gas chromatography cannot evaluate changes in each of the phospholipid fractions, including phosphatidylcholine, sphingomyelin, phosphatidylserine, phosphatidylethanolamine, sphingomyelin, phosphatidylserine, phosphatidylethanolamine, and phosphatidylinositol in the serum of the COVID-19 patients; it only gives us an approximation of the FA alterations in the TPLs in the serum of patients with COVID-19 and their modification by treatment with Vit E. To evaluate the specific changes, it would be necessary to separately evaluate each of the fractions of the aforementioned FAAIPF, such as thin-layer chromatography followed by HPLC-MS.

## Figures and Tables

**Table 1:** Demographic characteristics at admission in patients infected with COVID-19 before treatment with Vit E.

Variables	Median and Min–Max Range
Edad	55 (31–88)
Género	3 mujeres y 19 hombres
IMC	28 (20–38)
Comorbidity number and (%)	
Diabetes mellitus	6 (27)
Hipertensión	3 (14)
Dislipidemia	10 (46)
Peso normal	5 (23)
Exceso de peso	9 (41)
Obesidad mórbida	8 (36)
Gasometry and blood biochemistry median and min–max range	
PaO_2_	72 (47–233)
OCP _2_	32 (26–60)
PaO_2_ / FiO_2_ ( mmHg _)_	126 (50–318)
SPO_2_/FiO_2_ (mmHg)	144 (67–320)
HR bpm	85 (20–115)
MAP (mmHg)	74 (61–99)
Temperature	36 (35–38)
BUN	18.4 (8.6–40.9)
Leukocytes 10^3^/µL	9.5 (4.4–17.3)
Lymphocytes 10^3^/µL	6.5 (0.20–1.1)
Platelets 10^3^/µL	226 (97–529)
Ferritin ng/m	586 (189–1443)
D-dimer ng/dL	1040 (440–13,100)
N/L	12 (4–50)
Procalcitonin ng/dL	0.10 (0.02–10.7)
Median and Min–Max range	
SOFA	1 (0–8)
APACHE II	5.5 (3–8)
SAPS	28 (15–32)
Glasgow	15 (12–15)
CORADS	4 (3–5)
COVID GRAM	119 (76–175)

Abbreviations: BMI = body mass index, bpm = beats per minute, HR = heart rate, MAP = mean arterial pressure, N/L = neutrophils/lymphocytes. FiO_2_ = fraction of inspired oxygen, PaO_2_ = oxygen at arterial pressure, PCO_2_ = carbon dioxide at partial pressure, SPO_2_ = arterial oxygen saturation. SOFA = Organ Failure Sequential Assessment, APACHE = Acute Physiology and Chronic Health Assessment II, SAPS = Simplified Acute Physiology II Score. CORADS = A Categorical CT Assessment Scheme for Patients Suspected of Having COVID-19, COVID GRAM = COVID-19 Critical Illness Prediction Tool.

**Table 2:** Gender, age, and serum biochemical determinations in HSs and patients with COVID-19 before treatment with Vit E.

	HS (*n* = 23) Median Min-Max Range	COVID-19 (*n* = 22) Median Min-Max Range	*p*
Age	60 (27–87)	55 (31–88)	NS
Women	8 (35)	3 (14)	NS
Men	15 (55)	19 (86)	NS
Blood biochemical (mg/dL)			
Glucose	99 (32–199)	120 (81–330)	0.006
C^2+^	9.1 (8.4–9.7)	9.2 (7.9–10.10)	NS
Uric acid	6.6 (2.9–9.6)	4.7 (1.5–8.07)	0.01
Creatinine	0.83 (0.60–1.20)	0.90 (0.50–1.70)	NS
CT	165 (97–276)	134 (94–189)	0.01
HDL	37 (20–54)	34.7 (22.3–47.9)	NS
LDL	92 (30–148)	70.3 (38.9–129.7)	0.01
TG	115 (67–389)	122 (70–249)	NS

Abbreviations: C^2+^ = calcium, CT = cholesterol, HDL = high-density lipoprotein, LDL = low-density lipoprotein, TG = triglyceride.

**Table 3:** Inflammation markers in patients with COVID-19 before and after treatment with Vit E.

Variables	HSs	COVID-19 Patients Median (Min–Max)	COVID-19 Patients + Vit E Median (Min–Max)
IL-6 (pg/mL)	0.9 (0.4–1.8)	98.5 (7.8–637.6) ***	7.8 (7.8–547.7) *
CRP (mg/dL)	1.8 (0.2–35.0)	177 (4.1–384.7) ***	49 (1.8–173.5) *

HSs vs. COVID-19, *** *p* = 0.001, COVID-19 before vs. COVID-19 after plus Vit E * *p* = 0.01. Abbreviations: IL = interleukin, CRP = C-reactive protein, HSs = healthy subjects.

**Table 4:** Total fatty acid compositions in the serum in the HS and COVID-19 patients before and after treatment with Vit E.

Fatty Acid (%)	HSs	COVID-19 Patients	COVID-19 Patients + Vit E
C16:0	28.8 ± 1.5	24.9 ± 1.4 *	20.9 ± 1.3
C16:1	4.2 ± 0.3	3.1 ± 0.2 ***	4.0 ± 0.3 *
C18:0	12.0 ± 1.3	8.4 ± 0.4 *	9.1 ± 0.7
C18:1n-9	20.8 ± 0.8	26.9 ± 1.0 ***	24.9 ± 1.4
C18:2n-6	23.1 ± 1.3	22.8 ± 1.1	21.3 ± 2.0
γ-C18:3n-6	1.1 ± 0.3	1.4 ± 0.5	3.9 ± 1.9 **
α-C18:3n-6	1.0 ± 0.2	1.5 ± 0.3	3.2 ± 0.6 *
C20:3n-6	1.8 ± 0.6	1.8 ± 0.5	2.8 ± 0.4 **
C20:4n-6	3.5 ± 0.4	4.2 ± 0.5	5.8 ± 0.8
C20:5n-3	2.5 ± 0.5	2.2 ± 0.5	3.3 ± 0.4 ^§^
C22:6n-3	1.2 ± 0.3	1.6 ± 0.5	3.0 ± 0.6 **
Total fatty acid mg/mL in the serum			
	2.8 ± 0.2	3.2 ± 0.4 ^§^	2.0 ± 0.3 ^§^

Data represent the weights of each individual FA/weights of total FAs as percentages (mean ± standard error). Fatty acid nomenclature: C16:0, PA; C16:1n-7, palmitoleic acid; C18:0, stearic acid; C18:1n-9, OA; C18:2n-6, LA; γ-C18:3n-6, γ-linolenic acid; α-C18:3n-3, α-linolenic acid; C20:3n-6, D-γ-LA; C20:4n-6, AA; C20:5n-3 EPA; C22:6n-3, DHA. HSs vs. COVID-19 * *p* = 0.04, *** *p* = 0.001, COVID-19 before vs. COVID-19 after plus Vit E * *p* = 0.01, ** *p* = 0.02, ^§^ tendency = 0.08, ^§^ tendency = 0.09.

**Table 5:** Total fatty acid compositions of saturated, monounsaturated, and polyunsaturated fatty acids in the serum in HSs and COVID-19 patients before and after treatment with Vit E.

Fatty Acid (%)	HSs	COVID-19 Patients	COVID-19 Patients + Vit E
SFA	39.11 ± 2.20	32.69 ± 1.38 **	29.52 ± 1.45
MUFA	24.61 ± 0.76	30.04 ± 1.10 **	28.89 ± 1.25
PUFA (n-3)	30.27 ± 1.93	30.19 ± 1.12	33.82 ± 2.82 ^§^
PUFA (n-6)	5.77 ± 1.16	5.36 ± 1.06	9.52 ± 1.07 *

Data are represented as percentages (mean ± standard error). Abbreviations: SFA = saturated fatty acid, MUFA = monounsaturated fatty acid, PUFA = polyunsaturated fatty acid. Data are presented as the percentage of the mean ± SE. HSs vs. COVID-19 before * *p* = 0.01, ** *p* = 0.02, COVID-19 vs. COVID-19 after plus Vit E * *p* = 0.01, ^§^ tendency = 0.09.

**Table 6:** Indirect desaturation indexes of the desaturases in the total fatty acids from HS and COVID-19 patients before and after treatment with Vit E.

Desaturation Index	HSs	COVID-19 Patients	COVID-19 Patients + Vit E
C16:1n-7/C16:0 (∆^9^)	0.15 ± 0.008	0.13 ± 0.01 *	0.19 ± 0.01
C18:1n-9/C18:0 (∆^9^)	1.90 ± 0.10	3.36 ± 0.23 ***	2.97 ± 0.23
γ-C18:3n-6/C18:2n-6 (∆^6^)	3.02 ± 0.37	7.16 ± 2.01 **	2.29 ± 0.30 *
C20:4n-6/C20:3n-6 (∆^5^)	0.43 ± 0.09	0.62 ± 0.12	1.04 ± 0.19 **
C22:6n-3/C20:5n-3 (∆^5^)	0.05 ± 0.01	0.06 ± 0.02	0.33 ± 0.18 ^§^

Data are represented as percentages (mean ± standard error). Fatty acid nomenclature: C16:0, PA; C16:1n-7, palmitoleic acid; C18:0, stearic acid; C18:1n-9, OA; C18:2n-6, LA; γ-C18:3n-6, γ-linolenic acid; α-C18:3n-3, α-linolenic acid; C20:3n-6, D-γ-LA; C20:4n-6, AA; C20:5n-3 EPA; C22:6n-3, DHA. Data are presented as the percentage of the mean ± SE. HSs vs. COVID-19 before * *p* = 0.03, *** *p* = 0.001, COVID-19 before vs. COVID-19 after plus Vit E * *p* = 0.01, ** *p* = 0.001, ^§^ tendency = 0.07.

**Table 7:** Fatty acid compositions of the FAAIPF in the serum in the HSs before and after treatment with Vit E in COVID-19 patients.

Fatty Acid (%)	HSs	COVID-19 Patients	COVID-19 Patients + Vit E
C16:0	21.5 ± 1.2	28.4 ± 1.8 ***	23.6 ± 1.1 **
C16:1	3.3 ± 0.2	4.1 ± 0.5	4.5 ± 0.4
C18:0	7.8 ± 0.6	10.0 ± 0.6 *	9.8 ± 0.4
C18:1n-9	23.1 ± 1.3	20.0 ± 1.0 ^§^	17.7 ± 0.8
C18:2n-6	31.2 ± 1.2	20.4 ± 1.1 ***	21.9 ± 0.9
γ-C18:3n-6	0.9 ± 0.2	1.0 ± 0.2	1.4 ± 0.2
α-C18:3n-3	1.8 ± 0.6	1.2 ± 0.2	2.3 ± 0.9
C20:3n-6	2.7 ± 0.7	2.5 ± 0.2 ^§^	2.0 ± 0.6 *
C20:4n-6	4.7 ± 0.7	7.8 ± 1.5	7.4 ± 1.8
C20:5n-3	2.2 ± 0.8	2.4 ± 0.8	4.7 ± 0.9 *
C22:6n-3	0.9 ± 0.4	1.5 ± 0.5	4.4 ± 1.0 *

Data are represented as percentages (mean ± standard error). Fatty acid nomenclature: C16:0, PA; C16:1n-7, palmitoleic acid; C18:0, stearic acid; C18:1n-9, OA; C18:2n-6, LA; γ-C18:3n-6, γ-linolenic acid; α-C18:3n-3, α-linolenic acid; C20:3n-6, D-γ-LA; C20:4n-6, AA; C20:5n-3 EPA; C22:6n-3, DHA. Data are presented as the percentage of the mean ± SE. HS vs. COVID-19 before *** *p* = 0.002, HS vs. COVID-19 before * *p* = 0.01, HSs vs. COVID-19 before ^§^ *p* = 0.07, HSs vs. COVID-19 before *** *p* = 0.001, COVID-19 after plus Vit E vs. COVID-19 ** *p* = 0.03, COVID-19 after plus Vit E vs. COVID-19 before * *p* = 0.01.

**Table 8:** Fatty acid compositions of the FAAIPF, SFA, MUFA, and PUFA in the serum in the HS and COVID-19 patients before and after treatment with Vit E.

Fatty Acid (%)	HSs	COVID-19 Patients	COVID-19 Patients + Vit E
SFA	28.9 ± 0.9	38.4 ± 1.8 ***	33.4 ± 1.4 ^§^
MUFA	26.5 ± 1.2	24.2 ± 1.0	22.2 ± 0.8
PUFA (n-3)	39.6 ± 1.0	31.8 ± 1.3 ***	32.8 ± 1.4 ^§^
PUFA (n-6)	4.9 ± 1.5	5.6 ± 1.4	11.5 ± 2.0 *

Data are represented as percentages (mean ± standard error). Fatty abbreviations: SFA = saturated fatty acid, MUFA = monounsaturated fatty acid, PUFA = polyunsaturated fatty acid. Data are presented as the percentage of the mean ± SE. HSs vs. COVID-19 before *** *p* = 0.002, COVID-19 after plus vit E vs. COVID-19 before * *p* = 0.03, ^§^ HSs vs. COVID-19 before *p* = 0.08.

**Table 9:** Indirect desaturation indexes of the desaturases in the fatty acids of the FAAIPF from the HS and COVID-19 patients.

Desaturation Index	HSs	COVID-19 Patients	COVID-19 Patients + Vit E
C16:1n-7/C16:0 (∆^9^)	0.17 ± 0.02	0.16 ± 0.02	0.19 ± 0.01
C18:1n-9/C18:0 (∆^9^)	3.43 ± 0.37	2.13 ± 0.17 *	1.85 ± 0.12
γ-C18:3n-6/C18:2n-6 (∆^6^)	3.17 ± 0.72	4.00 ± 0.85	4.44 ± 0.89
C20:4n-6/C20:3n-6 (∆^5^)	0.28 ± 0.10	0.57 ± 0.24	0.68 ± 0.19 ^§^
C22:6n-3/C20:5n-3 (∆^5^)	0.03± 0.007	0.05 ± 0.01	0.07 ± 0.01 *

Data are represented as percentages (mean ± standard error). Fatty acid nomenclature: C16:0, PA; C16:1n-7, palmitoleic acid; C18:0, stearic acid; C18:1n-9, OA; C18:2n-6, LA; γ-C18:3n-6, γ-linolenic acid; α-C18:3n-3, α-linolenic acid; C20:3n-6, D-γ-LA; C20:4n-6, AA; C20:5n-3 EPA; C22:6n-3, DHA. Data are presented as the percentage of the mean ± SE. HSs vs. COVID-19 before * *p* = 0.03, ^§^ COVID-19 before vs. COVID-19 after plus *p* = 0.09.

## Data Availability

The datasets generated and analyzed during the current study are available from the corresponding author upon reasonable request.
